# Some convergently three-term trust region conjugate gradient algorithms under gradient function non-Lipschitz continuity

**DOI:** 10.1038/s41598-024-60969-9

**Published:** 2024-05-13

**Authors:** Wujie Hu, Jinzhao Wu, Gonglin Yuan

**Affiliations:** 1 School of Electrical Engineering, Guangxi University, Nanning, Guangxi People’s Republic of China; 2 School of Mathematics and Information Science, Center for Applied Mathematics of Guangxi (Guangxi University), Guangxi University, Nanning, Guangxi People’s Republic of China

**Keywords:** Conjugate gradient, Descent property, Trust region property, Gradient function non-Lipschitz continuity, Global convergence, Applied mathematics, Computational science

## Abstract

This paper introduces two three-term trust region conjugate gradient algorithms, TT-TR-WP and TT-TR-CG, which are capable of converging under non-Lipschitz continuous gradient functions without any additional conditions. These algorithms possess sufficient descent and trust region properties, and demonstrate global convergence. In order to assess their numerical performance, we compare them with two classical algorithms in terms of restoring noisy gray-scale and color images as well as solving large-scale unconstrained problems. In restoring noisy gray-scale images, we set the performance of TT-TR-WP as the standard, then TT-TR-CG takes around 2.33 times longer. The other algorithms around 2.46 and 2.41 times longer, respectively. In solving the same color images, the proposed algorithms exhibit relative good performance over other algorithms. Additionally, TT-TR-WP and TT-TR-CG are competitive in unconstrained problems, and the former has wide applicability while the latter has strong robustness. Moreover, the proposed algorithms are both more outstanding than the baseline algorithms in terms of applicability and robustness.

## Introduction

This paper considers following model

1 $$\begin{aligned} \min \{h(x)|~x\in R^n\}, \end{aligned}$$

where the objective function $h:R^n\longrightarrow R$ is continuously differentiable. The conjugate gradient (CG) algorithm is widely used to solve (1), in which the iteration formula is written as:

2 $$\begin{aligned} x_{k+1}=x_k+\alpha _kd_k, ~k=0,1,2,\ldots , \end{aligned}$$

where $x_{k+1},$ $\alpha _k$ and $d_k$ are next iteration point, step size and search direction respectively, where $d_k$ is generally defined by formula

3 $$\begin{aligned} d_k=\left\{ \begin{array}{ll} -g_k+\beta _{k}d_{k-1}, &{} \quad \text{ if }\,\,\,\, k\ge 1,\\ -g_k,&{} \quad \text{ if }\,\,\,\,k=0,\\ \end{array} \right. \end{aligned}$$

where $g_k$ is called the gradient of objective function *h*(*x*) at iteration point $x_k$, and $\beta _k\in R$ is a scalar. Some CG algorithms are proposed to solve large-scale optimization problems and engineer problems. In Ref.^4^, general conjugate gradient method using the Wolfe line search is proposed, with a condition on the scalar $\beta _k,$ which is sufficient for the global convergence. In Ref.^16^, a projection-based method is proposed to solve large-scale nonlinear pseudo-monotone equations, without Lipschitz continuity. In Refs.^19–21^, Sheng et al. proposed some trust region algorithms to solve nonsmooth minimization, large-residual nonsmooth least squares problems and optimization problems. Yuan et al proposed some nonlinear conjugate gradient methods to restore nonlinear equations and image restorations in Ref.^24,25^. In Ref.^5^, Dai summarized some analysis of conjugate gradient method. In Ref.^9^, authors adopted conjugate gradient solvers on graphic processing units. In Ref.^12^, authors proposed a new conjugate gradient method with guaranteed descent and an efficient line search for optimization. In Ref.^18^, authors proposed a hybrid conjugate gradient algorithm combining PRP and FR algorithms. In Ref.^23^, Wei et al proposed a conjugate gradient algorithm which designs a negative coefficient in the formula of the search direction. In fact, an important work is the design of $\beta _k,$ and some classical expressions are widely used, including the Hestenes-Stiefel (HS)^8,14,27^, Liu-Storey (LS)^22^, Polak-Ribière-Polyak (PRP)^11,25,26,28^, Dai-Yuan (DY)^6,29^ and conjugate descent method (CD)^10,13^, Fletcher-Reeves (FR)^15^, where the first three algorithms have relatively good numerical performance but fewer theoretical results, while the others are inverse. The definitions are listed in Table 1, where $\Vert .\Vert$ is the Euclidean norm.

**Table 1:** Six classical CG scalars.

	HS	LS	PRP	DY	CD	FR
$\beta _k$	$\frac{g_k^T(g_k-g_{k-1})}{(g_k-g_{k-1})^Td_{k-1}}$	$\frac{g_k^T(g_k-g_{k-1})}{-d_{k-1}^Tg_{k-1}}$	$\frac{g_k^T(g_k-g_{k-1})}{\Vert g_k\Vert ^2}$	$\frac{\Vert g_k\Vert ^2}{(g_k-g_{k-1})^Td_{k-1}}$	$\frac{\Vert g_k\Vert ^2}{ -d_{k-1}^Tg_{k-1}}$	$\frac{\Vert g_k\Vert ^2}{\Vert g_{k-1}\Vert ^2}$

The primary components of conjugate gradient algorithms encompass the search direction, step size (when applicable), and global convergence. The ultimate objective is to achieve a satisfactory balance between numerical efficiency and theoretical scrutiny.

In fact, the adequate descent property is a prerequisite for theoretical analysis and is governed by the following equation

4 $$\begin{aligned} g_k^Td_k\le -t\Vert g_k\Vert ^2, \end{aligned}$$

where $t>0.$ Moreover, the trust region technique illustrates that the search radius plays a crucial role in determining the numerical efficacy. The search direction is obtained by solving the subsequent quadratic function, where $\Delta _{k}$ denotes the trust region radius.

$$\begin{aligned}&\min _{x\in \Re ^n} g_{k}^{T}d_{k}+\dfrac{1}{2}d^{T}Q_{k}d.\\ & s.t.~~~\Vert d_{k}\Vert \le \Delta _{k}. \end{aligned}$$

The search direction in CG algorithms is also called satisfying the trust region property if following formula holds.

5 $$\begin{aligned} \Vert d_{k}\Vert \le t_1\Vert g_{k}\Vert , \end{aligned}$$

where $t_1 > 0$. Equations (4) and (5) are intimately connected with the global convergence. Furthermore, an inexact linear search approach is frequently utilized to determine a suitable step size $\alpha _k$. This paper adopts weak Wolfe-Powell (WWP) inexact linear search, which is formulated as follows:

6 $$\begin{aligned} h(x_k+\alpha _kd_k)\le h(x_k)+\delta \alpha _kg_k^Td_k \end{aligned}$$

and

7 $$\begin{aligned} g(x_k+\alpha _kd_k)^Td_k\ge \tau g_k^Td_k, \end{aligned}$$

where $$\delta \in (0,\frac{1}{2})$$ and $$\tau \in (\delta ,1)$$.

The aforementioned discussions are intricately linked to global convergence, which necessitates certain fundamental assumptions. These include: (i) the objective function must be continuously differentiable; (ii) the level set $S=\{x\in R^n: h(x)\le h(x_0)\}$ must be bounded; and (iii) the gradient function *g*(*x*) must be Lipschitz continuous, where $x_0$ denotes an initial point. The FR method^1^, modified HS method^7^, modified LS method^17^, and modified DY method^29^ achieve global convergence through the formula

$$\begin{aligned} \liminf _{k \rightarrow \infty } \Vert g_{k}\Vert =0. \end{aligned}$$

In other words, the Lipschitz continuity of the gradient function is a prerequisite for existing works, prompting us to consider whether global convergence can be attained in the absence of Lipschitz continuity. This paper proposes some three-term trust region conjugate gradient methods that converge under non-Lipschitz continuity condition, with the main properties summarized as follows:

- Objective algorithms possess both the sufficient descent and trust region properties, without any additional conditions. The trust region property is derived from the trust region algorithm, while the algorithm design is based on classical approaches such as Hestenes-Stiefel (HS) and Polak-Ribière-Polyak (PRP).
- These algorithms achieve global convergence even under conditions of non-Lipschitz continuity of the gradient function and weak Wolfe-Powell linear search techniques.
- The applications of these algorithms include image restoration of noisy gray scale and color images, as well as solving large-scale unconstrained problems. The case studies illustrate that TT-TR-WP and TT-TR-CG possess superior numerical performance.

The remainder of the paper is organized as follows: “Motivation and TT-TR-WP” provides an overview of the motivation behind TT-TR-WP; “The global convergence of TT-TR-WP” presents the convergence analysis; “TT-TR-CG and theoretical analysis” describes the TT-TR-WP algorithm and its convergence analysis; “Case studies” presents the case studies, including image restoration and large-scale unconstrained problem-solving; and finally, the last section offers concluding remarks.

## Motivation and TT-TR-WP

The first three-term conjugate gradient formula is proposed by Zhang et al.^30^, in which the search direction is defined by

8 $$\begin{aligned} d_k=\left\{ \begin{array}{ll} -g_k,&{} \text{ if }\,\,\,\,k=0,\\ -g_k+\frac{g_k^Ty_{k-1}d_{k-1}-g_k^Td_{k-1}y_{k-1}}{\Vert g_{k-1}\Vert ^2}, &{} \text{ if }\,\,\,\, k\ge 1.\\ \end{array} \right. \end{aligned}$$

Formula (8) satisfies the sufficient descent property without any additional conditions, while the trust region property is closely related to the objective function, Lipschitz continuity, and level set.

Formula (9) was introduced by Yuan et al.^28^ under the weak Wolfe-Powell linear search technique, where the search direction is given by the following expression:

9 $$\begin{aligned} d_k=\left\{ \begin{array}{ll} -g_k,&{} \text{ if }\,\,\,\,k=0,\\ -g_k+\alpha _{k-1}\frac{g_k^Ty_{k-1}d_{k-1}-g_k^Td_{k-1}y_{k-1}}{\Vert g_{k-1}\Vert ^2}, &{} \text{ if }\,\,\,\, k\ge 1,\\ \end{array} \right. \end{aligned}$$

The step size $\alpha _{k-1}$ is included in the search direction (9). This formula not only satisfies the sufficient descent property without other conditions, but also guarantees global convergence under non-Lipschitz continuity conditions, while the trust region property is closely linked to the formula $\alpha _{k-1}d_{k-1} = x_{k} - x_{k-1}$, objective function, and level set.

To summarize, while formulas (8) and (9) do possess the sufficient descent property without additional conditions, there are several limitations. The trust region property, vital for both theoretical analysis and numerical performance, unfortunately depends on the objective function, basic assumptions, and complex analysis. Additionally, there exist simpler and more cost-effective algorithms that simultaneously achieve better numerical performance and theoretical results.

Aforementioned discussions inspire us to propose following formula.

10 $$\begin{aligned} d_k=\left\{ \begin{array}{ll} -g_k,&{} \text{ if }\,\,\,\,k=0,\\ -g_k+\frac{g_k^Ty_{k-1}d_{k-1}-g_k^Td_{k-1}y_{k-1}}{\sigma \Vert d_{k-1}\Vert \Vert y_{k-1}\Vert +|d_{k-1}y_{k-1}|}, &{} \text{ if }\,\,\,\, k\ge 1,\\ \end{array} \right. \end{aligned}$$

### Remark 1

(i) Formula (10) possesses the sufficient descent and trust region properties that are independent of any additional conditions.
(ii) Global convergence is guaranteed even under conditions of non-Lipschitz continuity of the gradient function.
(iii) The classical HS algorithm’s excellent numerical performance is incorporated into TT-TR-WP through a specified denominator.

This section presents Algorithm 1, while the subsequent section provides the theoretical analysis.

**TT-TR-WP**: A convergently three-term trust region algorithm with the weak Wolfe-Powell linear search

- Step 0: Initialize $x_0\in R^n$, $d_0=-g_0$, constants $\epsilon \in (0,1)$, $\delta \in (0,\frac{1}{2})$, $\tau \in (\delta ,1)$, $\sigma >0$, and set $k=0$.
- Step 1: Stop rule $\Vert g_k\Vert \le \epsilon$.
- Step 2: Choose step size $\alpha _k$ under formulas (6) and (7).
- Step 3: Update iteration point $x_{k+1} = x_k+\alpha _kd_k$.
- Step 4: Stop rule $\Vert g_{k+1}\Vert \le \epsilon$.
- Step 5: Update search direction under formula (10).
- Step 6: Set $k=k+1$, and go to Step 2.

## The global convergence of TT-TR-WP

This section analyzes the global convergence of TT-TR-WP, in which the properties of sufficient descent and trust region are firstly given.

### Lemma 3.1

*The search direction* (10) *simultaneously has the sufficient descent* (4) *and trust region* (5) *properties*, *i.e.*,

11 $$\begin{aligned} g_k^Td_k = -\Vert g_k\Vert ^2, \end{aligned}$$

*and*

12 $$\begin{aligned} \Vert d_k\Vert \le (1+\frac{2}{\sigma })\Vert g_k\Vert , \end{aligned}$$

### Proof

If $$k=0$$, $$d_0=-g_0,$$ and $$\Vert d_0\Vert \le \Vert g_0\Vert \le (1+\frac{2}{\sigma })\Vert g_0\Vert ,$$

If $k\ge 1$, following formulas can be obtained from the formula (10):

$$\begin{aligned} g_k^Td_k= & {} g_k^T\left( -g_k+\frac{g_k^Ty_{k-1}d_{k-1}-g_k^Td_{k-1}y_{k-1}}{\sigma \Vert d_{k-1}\Vert \Vert y_{k-1}\Vert +|d_{k-1}y_{k-1}|}\right) \\= & {} -\Vert g_k\Vert ^2+g_k^T\frac{g_k^Ty_{k-1}d_{k-1}-g_k^Td_{k-1}y_{k-1}}{\sigma \Vert d_{k-1}\Vert \Vert y_{k-1}\Vert +|d_{k-1}y_{k-1}|}\\= & {} -\Vert g_k\Vert ^2. \end{aligned}$$

and

$$\begin{aligned} \Vert d_k\Vert= & {} \Vert -g_k+\frac{g_k^Ty_{k-1}d_{k-1}-g_k^Td_{k-1}y_{k-1}}{\sigma \Vert d_{k-1}\Vert \Vert y_{k-1}\Vert +|d_{k-1}y_{k-1}|}\Vert \\\le & {} \Vert g_k\Vert +\frac{2\Vert g_k\Vert \Vert y_{k-1}\Vert \Vert d_{k-1}\Vert }{\sigma \Vert d_{k-1}\Vert \Vert y_{k-1}\Vert +|d_{k-1}y_{k-1}|}\\\le & {} (1+\frac{2}{\sigma })\Vert g_k\Vert , \end{aligned}$$

then completes the proof. $\square$

### Remark 2

(i) The Lemma 3.1 proves the sufficient descent and trust region properties of search direction (10), which are independent of any assumptions and linear search techniques.
(ii) From formula (11), we can obtain
  $$\begin{aligned} -\Vert d_k\Vert \Vert g_k\Vert \le g_k^Td_k = -\Vert g_k\Vert ^2, \end{aligned}$$
  this means that
  $$\begin{aligned} \Vert g_k\Vert \le \Vert d_k\Vert , \end{aligned}$$
  thus following formula holds from formula (12)
  13 $$\begin{aligned} \Vert g_k\Vert \le \Vert d_k\Vert \le (1+\frac{2}{\sigma })\Vert g_k\Vert ,\forall \, k. \end{aligned}$$

To achieve global convergence, certain basic assumptions are proposed.

### Assumption

(i) The level set $S=\{x| h(x)\le h(x_0)\}$ is well-defined and bounded, where $x_0$ is the initial point.
(ii) The function *h*(*x*) is continuously differentiable and bounded below.

Under these assumptions, the following significant properties hold:

**Property 1:** The iteration sequence $\{x_k\}$ is bounded.

**Property 2:** The gradient function *g*(*x*) is continuous on the level set.

Now pay attention to the global convergence of TT-TR-WP.

### Theorem 3.1

*If sequences* $\{x_k,d_k,\alpha _k,g_k\}$ *are generated by TT-TR-WP*, *then, following formula holds*

14 $$\begin{aligned} \liminf _{k \rightarrow \infty } \Vert g_{k}\Vert =0. \end{aligned}$$

### Proof

We adopt proof by contradiction, and firstly make an assumption

15 $$\begin{aligned} \Vert g_k\Vert \ge \varepsilon _C, \end{aligned}$$

where $\varepsilon _C$ is a positive constant.

Additionally, there exists a convergent subsequence $\{x_{k_i}\}$ since iteration point $\{x_k\}$ is bounded, it means that

$$\begin{aligned} x_{k_i}\rightarrow x^*, i\rightarrow \infty , \end{aligned}$$

Similarly, the gradient function is continuous, thus there exists $\epsilon _1>0$ and an integer $N_1>0$ such that

16 $$\begin{aligned} \Vert g(x_{k_i})-g(x^*)\Vert <\epsilon _1,\,\,\forall \,\,i>N_1. \end{aligned}$$

From formula (13), there exists $\epsilon _2>0,$ and an integer $N_2>0$ satisfying

17 $$\begin{aligned} \Vert d(x_{k_i})-d(x^*)\Vert <\epsilon _2,\,\,\forall \,\,i>N_2. \end{aligned}$$

From (16), (17) and (11), following formula holds

18 $$\begin{aligned} g(x^*)^Td(x^*)\le -\Vert g(x^*)\Vert ^2 \le -\varepsilon _C^2<0. \end{aligned}$$

On the other hand, following formula will be obtained from (7)

$$\begin{aligned} g(x_k+\alpha _kd_k)^Td_k\ge \tau g_k^Td_k, \end{aligned}$$

thus

$$\begin{aligned} g_{k_{i+1}}^Td_{k_{i}} - \tau g_{k_{i}}^Td_{k_{i}}\ge 0, \end{aligned}$$

then taking the limit on both sides and set $N=\max \{N_1,N_2\},$ with the subsequence $\{x_{k_i}\},$ we can deduce that

$$\begin{aligned} \lim _{i \rightarrow \infty }(g_{k_{i+1}}^Td_{k_{i}}-\tau g_{k_{i}}^Td_{k_{i}})=(1-\tau )g(x^*)^Td(x^*)\ge 0. \end{aligned}$$

It means that there exists a subsequence $\{x_{k_i}\},$ such that

$$\begin{aligned} g(x^*)^Td(x^*)\ge 0, \end{aligned}$$

while this contradicts the relation (11), i.e. the original formula holds and the proof is completed. $\square$

### Remark 3

(i) Non-Lipschitz continuous gradient functions are prevalent. For instance, $g(x) = \sin (\frac{1}{x})$ and $g(x)=x^{\frac{3}{2}}\sin (\frac{1}{x})$ for $x\in (0, 1].$
(ii) The global convergence of TR-TR-WP is established under the weak Wolfe-Powell linear search technique and gradient function non-Lipschitz continuity.
(iii) The sufficient descent and trust region properties, (11) and (12), simplify the convergence analysis.

## TT-TR-CG and theoretical analysis

This section will propose the other modified three-term trust region CG algorithm, TT-TR-CG, and prove some properties.

In TT-TR-CG, the search direction has following form:

19 $$\begin{aligned} d_k=\left\{ \begin{array}{ll} -g_k,&{} \text{ if }\,\,\,\,k=0,\\ -g_k+\frac{g_k^Ty_{k-1}d_{k-1}-g_k^Td_{k-1}y_{k-1}}{max\{\mu \Vert d_{k-1}\Vert \Vert y_{k-1}\Vert ,\Vert g_{k-1}\Vert ^2\}}, &{} \text{ if }\,\,\,\, k\ge 1,\\ \end{array} \right. \end{aligned}$$

where $$\mu > 0.$$

This subsection will firstly describe contents of objective algorithm.

TT-TR-CG: A convergently three-term trust region CG with the weak Wolfe-Powell

- Step 0: Initialize $x_0\in R^n$, $d_0=-g_0$, constants $\epsilon \in (0,1)$, $\delta \in (0,\frac{1}{2})$, $\tau \in (\delta ,1)$, $\mu >0$, and set $k=0$.
- Step 1: Stop rule $\Vert g_k\Vert \le \epsilon$.
- Step 2: Choose step size $\alpha _k$ under formulas (6) and (7).
- Step 3: Update iteration point $x_{k+1} = x_k+\alpha _kd_k$.
- Step 4: Stop rule $\Vert g_{k+1}\Vert \le \epsilon$.
- Step 5: Update search direction under formula (19).
- Step 6: Set $k=k+1$, and go to Step 2.

### Remark 4

(i) The search direction (19) satisfies both the sufficient descent and trust region properties simultaneously.
(ii) Global convergence analysis is established under the gradient function non-Lipschitz continuity and weak Wolfe-Powell linear search technique.
(iii) The good numerical performance of the classical PRP algorithm is partly incorporated into TT-TR-CG through the specified denominator.

### Lemma 4.1

*The search direction* (19) *has the sufficient descent* (4) *and trust region* (5) *properties simultaneously without any conditions*, *i.e.*,

20 $$\begin{aligned} g_k^Td_k = -\Vert g_k\Vert ^2, \end{aligned}$$

*and*

21 $$\begin{aligned} \Vert d_k\Vert \le (1+\frac{2}{\mu })\Vert g_k\Vert . \end{aligned}$$

### Proof

The proof is similar with the TT-TR-WP, thus omits it. $\square$

To obtain the global convergence, some basic assumptions are proposed.

### Assumption

(i) the level set $S=\{x| h(x)\le h(x_0)\}$ is defined and bounded, where $x_0$ is an initial point;
(ii) the objective function *h*(*x*) is continuously differentiable and bounded below.

### Theorem 4.1

*If sequences* $\{x_k,d_k,\alpha _k,g_k\}$ *are generated by TT-TR-CG, then, following formula holds*

22 $$\begin{aligned} \liminf _{k \rightarrow \infty } \Vert g_{k}\Vert =0. \end{aligned}$$

### Proof

The proof is similar with the “The global convergence of TT-TR-WP”, then completes the proof. $\square$

## Case studies

This section utilises objective algorithms to restore noisy images and solve large-scale unconstrained optimisation problems to test their numerical performance.

To further test the numerical performance, this paper introduces two baseline algorithms in Ref.^26,28^, namely MPRP and A-TPRP-A, and the formulas are (8), (9), respectively. The former is the first three-term conjugate gradient algorithm and is widely cited. The latter is the latest algorithm which updates the search direction with the step size and possesses global convergence without Lipschitz continuity. The baseline algorithms possess both good numerical performance and theoretical properties in the existing works.

The experimental environment consists of an Intel(R) Core(TM) i5-8250U CPU @ 1.60GHz 1.80 GHz with 16 GB RAM running on the Windows 11 operating system.

### Image restoration

The restoration of noisy images is of great practical importance and is widely used. This subsection uses the TT-TR-WP, TT-TR-CG and baseline algorithms to restore noisy images to test their numerical performance, in which three figures are chosen because they are widely used and classical test figures, see Refs.^24,25^.

The objective function and experimental settings are described as follows: The candidate noise index set is denoted as *N*, the objective function as $\omega (u)$, and the edge-preserving function as $\chi$. The true image containing $K\times L$ pixels is denoted as *x*. For a more detailed explanation of image restoration, please refer to Refs.^3,24,25,28^.

$$\begin{aligned} N:=\left\{ (i, j) \in I \mid {\bar{\zeta }}_{i, j} \ne \zeta _{i, j}, \zeta _{i, j}=s_{\min } \text{ or } s_{\max }\right\} , \end{aligned}$$

where $I = \{1, 2, \ldots , K\} \times \{1,2,\ldots ,L,\},$ $\zeta _{i, j}$ is the observed noisy image and ${\bar{\zeta }}_{i, j}$ is the verified image, $s_{min}$ and $s_{max}$ are the minimum and maximum noisy pixel. Consider following optimization function

$$\begin{aligned} \min _u \omega (u) \end{aligned}$$

and

$$\begin{aligned} \omega (u)=\sum _{(i, j) \in N}\left\{ \sum _{(m, n) \in \phi _{i, j} \backslash N} \chi \left( u_{i, j}-\zeta _{m, n}\right) +\frac{1}{2} \sum _{(m, n) \in \phi _{i, j} \bigcap {N}} \chi \left( u_{i, j}-u_{m, n}\right) \right\} , \end{aligned}$$

$$\phi _{i, j} = \{(i,j-1), (i,j+1),(i-1,j),(i+1,j)\}.$$

$$\begin{aligned} \chi =\left\{ \begin{array}{ll} t^{2} / \nu , &{} \text{ if } \quad |t| \le \nu \\ |t|-2 \nu , &{} \text{ if } \quad |t|>\nu , \end{array}\right. \end{aligned}$$

where $$\nu > 0.$$

$$\begin{aligned} PSNR=10\times \log _{10}\left( \frac{(2^{num}-1)^2}{MSE}\right) , \end{aligned}$$

where MSE is the mean square error between the original image and processed image and *num* is the number of bits.

The stop rule of algorithm is $\frac{\Vert h_{k+1}-h_k\Vert }{\Vert h_k\Vert }<\varepsilon$, and the parameters are $\delta =0.2, \tau = 0.895, \sigma =0.1, \mu = 0.1, \varepsilon =10^{-6}.$

**Table 2:** The running time under different noise ratios with diverse algorithms.

Figure	Noise ratio	TT-TR-WP	TT-TR-CG	A-T-PRP-A	MPRP
Baboon	0.2	8.52	13.48	12.45	13.66
	0.5	12.09	31.23	28.97	29.97
	0.7	28.11	44.77	41.94	45.77
	0.9	25.64	65.33	64.88	75.34
Barbara	0.2	7.11	9.77	10.31	9.33
	0.5	15.00	26.44	28.16	28.78
	0.7	15.00	38.56	44.66	42.17
	0.9	38.81	66.09	89.39	77.28
Man	0.2	27.25	47.22	39.98	52.19
	0.5	47.45	96.67	116.52	104.69
	0.7	76.38	168.45	171.92	190.13
	0.9	97.83	332.23	339.77	306.75
Cameraman	0.2	1.86	1.98	3.30	3.53
	0.5	2.27	6.03	6.77	6.94
	0.7	4.36	8.61	10.42	8.61
	0.9	6.16	13.59	14.66	15.36
Boat	0.2	1.52	3.38	4.91	1.67
	0.5	2.83	4.83	5.73	4.52
	0.7	3.08	8.30	6.95	5.58
	0.9	5.70	10.89	10.45	10.44

**Table 3:** The ratio of total running time comparing with TT-TR-WP.

Figure	TT-TR-WP	TT-TR-CG	A-T-PRP-A	MPRP
Baboon	1.00	2.08	1.99	2.22
Barbara	1.00	1.86	2.27	2.08
Man	1.00	2.59	2.68	2.63
Cameraman	1.00	2.06	2.40	2.35
Boat	1.00	2.09	2.14	1.69
All figures	1.00	2.34	2.46	2.42

**Table 4:** The SSIM and PSNR under different noise ratios with diverse algorithms.

Figure	Noise ratio	SSIM				PSNR			
		TT-TR-WP	TT-TR-CG	A-T-PRP-A	MPRP	TT-TR-WP	TT-TR-CG	A-T-PRP-A	MPRP
Baboon	0.2	0.93	0.93	0.93	0.93	29.44	29.45	29.39	29.35
	0.5	0.78	0.78	0.78	0.78	24.57	24.57	24.54	24.51
	0.7	0.61	0.61	0.61	0.61	22.35	22.34	22.31	22.34
	0.9	0.31	0.31	0.31	0.31	20.31	20.25	20.29	20.28
Barbara	0.2	0.94	0.94	0.94	0.94	31.13	31.12	31.13	31.01
	0.5	0.81	0.81	0.81	0.81	26.33	26.36	26.34	26.39
	0.7	0.68	0.68	0.68	0.68	24.50	24.55	24.52	24.52
	0.9	0.44	0.44	0.44	0.44	22.54	22.50	22.46	22.51
Man	0.2	0.93	0.93	0.93	0.93	38.02	38.00	37.96	37.90
	0.5	0.81	0.81	0.81	0.81	32.51	32.49	32.54	32.50
	0.7	0.68	0.68	0.68	0.68	29.42	29.40	29.49	29.44
	0.9	0.41	0.41	0.41	0.41	25.28	25.21	25.28	25.30
Cameraman	0.2	0.93	0.93	0.93	0.93	32.26	32.60	32.59	32.21
	0.5	0.78	0.78	0.78	0.78	27.13	27.28	27.55	27.45
	0.7	0.63	0.62	0.62	0.62	24.76	24.69	24.57	24.68
	0.9	0.32	0.32	0.33	0.32	21.10	20.88	21.09	20.95
Boat	0.2	0.94	0.94	0.94	0.94	32.51	32.15	32.31	32.40
	0.5	0.80	0.80	0.80	0.80	27.16	27.29	27.13	27.00
	0.7	0.64	0.64	0.65	0.64	24.57	24.52	24.65	24.48
	0.9	0.36	0.36	0.36	0.37	21.59	21.61	21.46	21.74

In restoring noisy gray-scale images, from Table 2, we can conclude that TT-TR-WP exhibits the best numerical performance in terms of running time, TT-TR-CG is the second best, MPRP is third, and A-T-PRP-A is the slowest. Furthermore, if we set the performance of TT-TR-WP as the standard, then TT-TR-CG takes around 2.34 times longer. The other algorithms take around 2.46 and 2.42 times longer, respectively. In Table 3, the time proportion among all algorithms in each figure and all figures is proposed, in which the biggest gap is 1.68, TT-TR-WP is far ahead than the others, and TT-TR-CG is pretty good in most situations. Additionally, results in Table 4 further demonstrate that all algorithms obtain highly similar SSIM and PSNR values. Combining the above discussion, we can make a conclusion: to obtain highly similar results, TT-TR-WP and TT-TR-CG perform relatively well and the proposed algorithms are competitive.

**Figure 1:**
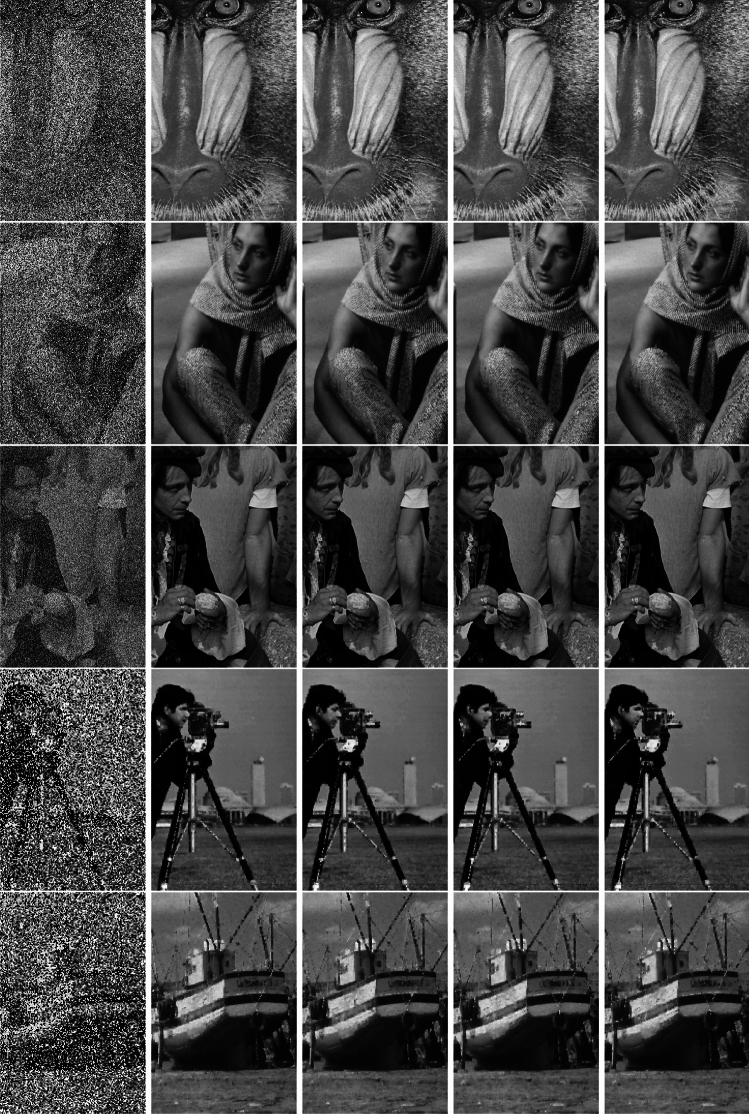
From left to right, the images disturbed by 50$\%$ salt-and-pepper noise, the images restored by TT-TR-WP (first column), TT-TR-CG (second column), A-T-PRP-A (third column) and MPRP (last column), respectively.

In summary, TT-TR-WP exhibits impressive numerical performance, and TT-TR-CG is highly competitive with the others. To save space, this paper only records numerical results but abandons the display of figures obtained by diverse algorithms with noise ratios of 70%, and 90%, see Fig. 1. In each row, the first column is obtained by TT-TR-WP, the second column by TT-TR-CG, the third column by A-T-PRP-A, and the last column by MPRP.

### Color image restoration

To further evaluate the performance of the objective algorithms, this section applies various algorithms to restore color images with different levels of noise. Peak signal-to-noise ratio (PSNR) and structural similarity index measure (SSIM) and Mean Squared Error (MSE) are widely used measurements for image quality assessment and are used in this section. To save space, this paper only records numerical results but abandons the display of figures obtained by diverse algorithms with noise ratios of 20%, 60% and 80%. The stop rule of algorithm is $\frac{\Vert h_{k+1}-h_k\Vert }{\Vert h_k\Vert }<\varepsilon$, and the parameters are $\delta =0.0885, \tau = 0.885, \sigma =0.0015, \mu = 1.1555, \varepsilon =10^{-4}.$

**Table 5:** The running time with different noise ratios across various algorithms.

Figure	Ratio	TT-TR-WP	TT-TR-CG	A-T-PRP-A	MPRP
car1	0.2	1.09	1.13	1.16	1.36
	0.4	1.39	1.34	1.55	1.36
	0.6	1.94	2.03	2.13	1.86
	0.8	4.91	4.59	4.72	4.72
l1ama	0.2	6.84	7.28	8.58	7.41
	0.4	9.70	10.17	10.78	9.80
	0.6	11.80	12.42	13.00	12.36
	0.8	19.58	19.63	22.47	19.48
fabricu	0.2	1.53	1.22	1.58	1.14
	0.4	1.64	1.56	1.50	1.56
	0.6	2.08	1.83	1.91	1.97
	0.8	3.25	3.81	2.95	2.98
car2	0.2	1.11	1.13	1.06	1.02
	0.4	1.47	1.42	1.28	1.39
	0.6	1.75	1.56	1.80	2.03
	0.8	3.75	3.75	3.56	4.08
All figures	Total sum	73.83	74.88	80.02	74.52

**Figure 2:**
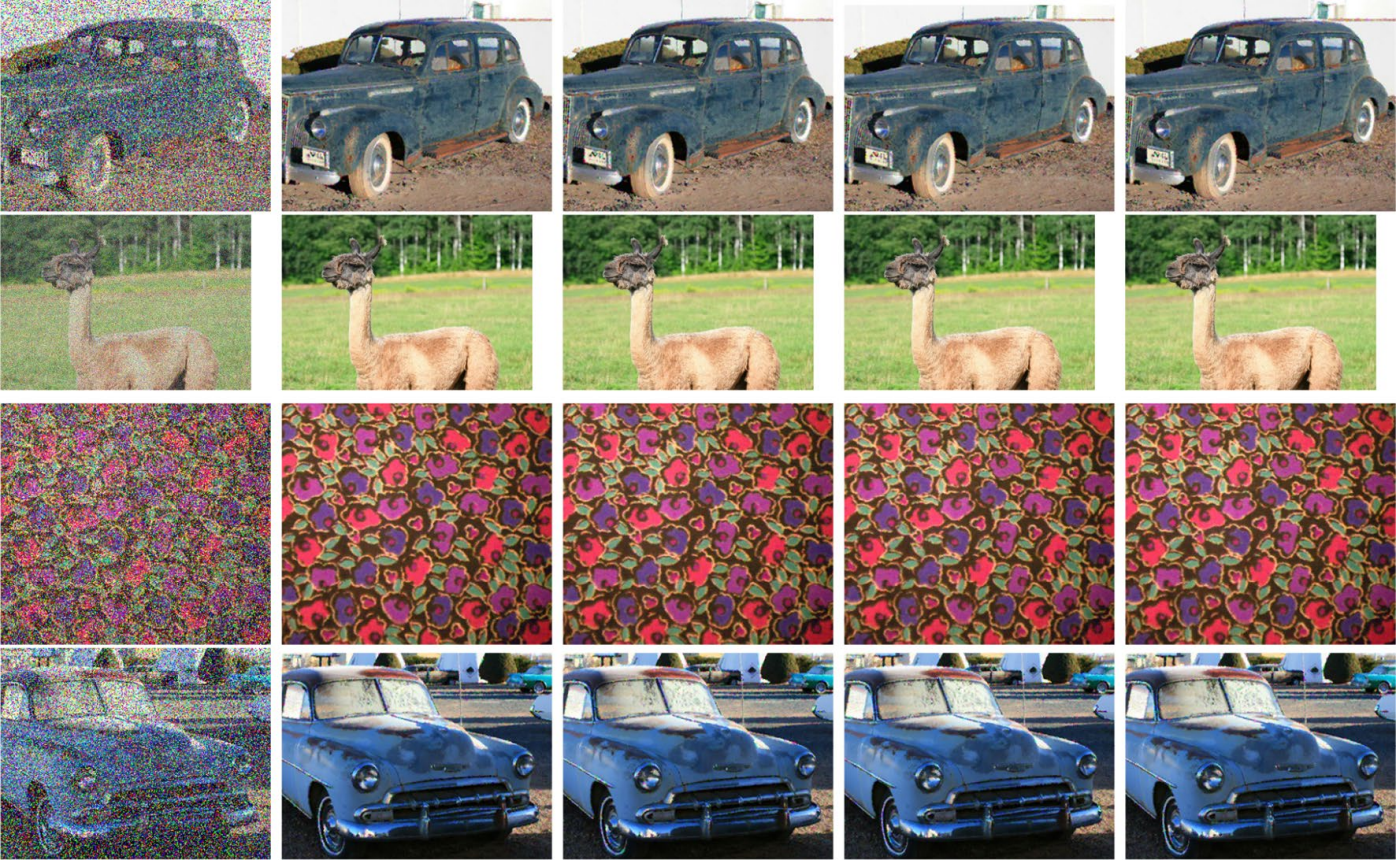
From left to right, the images disturbed by 40$\%$ salt-and-pepper noise, the images restored by TT-TR-WP (first column), TT-TR-CG (second column), A-T-PRP-A (third column) and MPRP (last column), respectively.

In Table 5, the total running time of four algorithms is 73.83, 74.88, 80.02, 74.52 s, respectively. Additionally, from Tables 6, 7, 8, the PSNR, MSE, and SSIM of algorithms are highly similar, but object algorithms are relatively competitive. The images restored by various algorithms under different noise ratios are presented in Fig. 2 that corresponds to noise ratio 40%. In each row, the first column is obtained by TT-TR-WP, the second column by TT-TR-CG, the third column by A-T-PRP-A, and the last column by MPRP.

**Table 6:** The PSNR with different noise ratios across various algorithms.

Figure	Ratio	TT-TR-WP	TT-TR-CG	A-T-PRP-A	MPRP
car1	0.2	29.36	29.32	29.27	29.24
	0.4	25.25	25.16	25.12	25.14
	0.6	22.32	22.31	22.32	22.31
	0.8	19.58	19.59	19.57	19.58
l1ama	0.2	39.49	39.40	39.44	39.49
	0.4	35.00	35.02	35.00	35.01
	0.6	31.71	31.71	31.69	31.72
	0.8	28.27	28.23	28.25	28.22
fabricu	0.2	31.01	30.95	30.93	30.90
	0.4	26.00	26.01	26.02	25.98
	0.6	22.35	22.35	22.31	22.30
	0.8	18.67	18.65	18.62	18.64
car2	0.2	29.77	29.82	29.84	29.72
	0.4	25.35	25.31	25.34	25.38
	0.6	22.26	22.19	22.14	22.28
	0.8	19.15	19.16	19.21	19.15
All figures	Total sum	425.53	425.15	425.05	425.07

**Table 7:** The MSE with different noise ratios across various algorithms.

Figure	Ratio	TT-TR-WP	TT-TR-CG	A-T-PRP-A	MPRP
car1	0.2	75.32	75.98	76.97	77.38
	0.4	194.34	198.09	200.18	198.95
	0.6	381.36	381.88	381.10	382.07
	0.8	716.74	714.57	717.15	716.70
l1ama	0.2	7.31	7.47	7.40	7.32
	0.4	20.56	20.48	20.57	20.52
	0.6	43.87	43.87	44.11	43.78
	0.8	96.93	97.82	97.37	98.00
fabricu	0.2	51.53	52.30	52.53	52.80
	0.4	163.23	163.11	162.48	163.95
	0.6	378.65	378.81	382.11	382.49
	0.8	882.58	888.25	894.08	890.33
car2	0.2	68.57	67.75	67.52	69.33
	0.4	189.73	191.64	190.02	188.32
	0.6	386.18	393.05	397.19	384.83
	0.8	790.57	789.40	780.23	790.19
All figures	Total sum	4447.48	4464.48	4471.02	4466.96

**Table 8:** The SSIM with different noise ratios across various algorithms (s).

Figure	Ratio	TT-TR-WP	TT-TR-CG	A-T-PRP-A	MPRP
car1	0.2	0.963	0.963	0.962	0.962
	0.4	0.904	0.903	0.902	0.902
	0.6	0.816	0.816	0.816	0.814
	0.8	0.679	0.677	0.679	0.679
l1ama	0.2	0.996	0.996	0.996	0.996
	0.4	0.988	0.988	0.988	0.988
	0.6	0.974	0.974	0.974	0.975
	0.8	0.946	0.946	0.946	0.946
fabricu	0.2	0.984	0.984	0.984	0.984
	0.4	0.950	0.950	0.950	0.950
	0.6	0.886	0.886	0.885	0.885
	0.8	0.746	0.747	0.745	0.747
car2	0.2	0.961	0.962	0.961	0.961
	0.4	0.898	0.898	0.899	0.898
	0.6	0.807	0.806	0.805	0.807
	0.8	0.661	0.659	0.662	0.662
All figures	Total sum	14.158	14.153	14.152	14.153

### General unconstrained optimization

To further test the numerical performance, this subsection applies the algorithms to solve large-scale unconstrained optimization problems. Sixty-five classical functions are randomly selected from^2^, as shown in Table 9, with dimensions of 3000, 6000, and 12,000. The stopping criterion is $\Vert g(x_k)\Vert <\varepsilon$ or $NI > 8000$, where *NI* is the iteration number, and $g(x_k)$ is the gradient value at the point $x_k$. The parameters used are $\delta =0.2, \tau = 0.9, \sigma =0.001, \mu = 0.1, \varepsilon =10^{-6}$.

**Table 9:** Test functions.

Function	Function	Function	Function	Function
EG2	LIARWHD	Extended PSC1	Extended Himmelblau	ENGVAL1
Hager	EDENSCH	Quadratic QF1	Extended tridiagonal-1	FLETCHCR
TRIDIA	DIXMAANE	Extended wood	Generalized tridiagonal-1	DIXMAANI
VARDIM	ARWHEAD	Extended Hiebert	Generalized Tridiagonal-2	DIXON3DQ
Raydan 1	Diagonal 1	Extended Powell	Partial perturbed quadratic	DIXMAANK
Raydan 2	Diagonal 3	Generalized PSC1	Almost perturbed quadratic	DIXMAANL
NONDIA	DIXMAANA	Extended penalty	Extended block diagonal BD1	DIXMAAND
BDQRTIC	DIXMAANB	Extended maratos	Quadratic diagonal perturbed	DIXMAANF
DQDRTIC	DIXMAANC	Perturbed quadratic	Tridiagonal perturbed quadratic	DIXMAANG
Diagonal 4	Extended Cliff	Extended Rosenbrock	Extended Freudenstein and Roth	DIXMAANH
Diagonal 5	NONDQUAR	Extended tridiagonal-2	Extended three exponential terms	DIAGONAL 6
Extended Trigonometric	Extended EP1	Extended DENSCHNB	Extended quadratic penalty QP1	STAIRCASE S1
Extended White and Holst	Extended Beale	Extended DENSCHNF	Extended quadratic penalty QP2	Broyden tridiagonal

**Figure 3:**
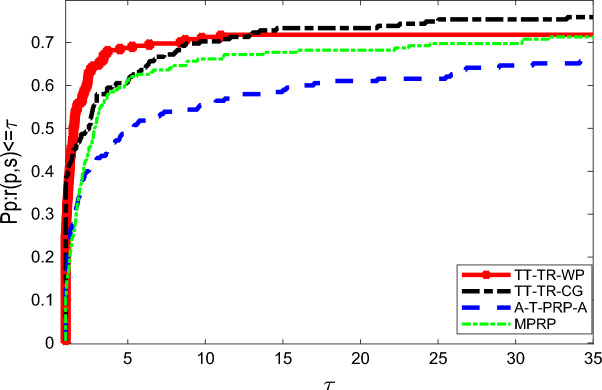
The running time of diverse algorithms on tested problems.

The running time in seconds is used as the reference standard for evaluating numerical performance, as shown in Table 10. The relative numerical performance of solving large-scale problems is illustrated in Fig. 3, in which the red line denotes TT-TR-WP, black line denotes TT-TR-CG, blue line denotes A-T-PRP-A, and the other denotes MPRP. TT-TR-WP has a high initial value, which means that possesses relatively good robustness. TT-TR-CG exhibits gradually increase trend all time which means that possesses relatively good applicability. TT-TR-WP and TT-TR-CG both possess relatively good robustness and applicability than the others.

In summary, TT-TR-WP and TT-TR-CG possess relatively good numerical performance than baseline algorithms, in terms of applicability and robustness, in which TT-TR-WP has the best robustness and relatively good applicability and TT-TR-CG is the opposite.

**Table 10:** The running time of diverse algorithms on tested problems.

No	TT-TR-WP			TT-TR-CG			A-T-PRP-A			MPRP		
	3000	6000	12,000	3000	6000	12,000	3000	6000	12,000	3000	6000	12,000
1	0.06	1.61	2.06	0.77	1.55	9.44	0.63	44.86	45.39	0.17	17.28	1.78
2	0.03	0.14	0.31	0.11	0.95	0.09	0.03	0.25	2.80	0.05	0.31	2.08
3	0.42	8.25	37.94	0.05	0.00	0.00	0.59	9.89	41.25	0.56	8.69	41.80
4	0.02	1.48	1.66	0.06	0.33	1.03	0.06	1.91	7.30	0.02	0.17	1.05
5	0.13	2.56	13.31	0.39	9.23	37.78	0.23	4.81	35.63	0.23	5.06	26.75
6	0.00	0.00	0.27	0.00	0.00	0.17	0.02	0.05	0.19	0.00	0.05	0.27
7	0.00	0.05	0.88	0.00	0.09	0.00	0.55	7.95	41.03	0.00	0.00	0.34
8	3.64	57.22	188.64	2.30	25.13	54.69	15.33	90.16	312.77	4.69	79.83	750.31
9	0.00	0.05	0.47	0.00	0.13	0.13	0.02	0.28	0.95	0.02	0.09	1.14
10	0.00	0.00	0.00	0.00	0.00	0.00	0.00	0.00	0.09	0.00	0.00	0.00
11	0.00	0.05	0.09	0.00	0.00	0.02	0.02	0.00	0.19	0.02	0.00	0.19
12	0.11	0.64	2.95	0.05	0.52	2.56	0.09	0.50	2.80	0.08	0.70	2.72
13	0.17	0.84	2.80	0.17	0.66	2.34	11.25	74.19	265.77	0.27	1.19	4.06
14	0.00	0.00	0.34	0.05	0.17	0.64	0.58	9.05	47.61	0.00	0.11	0.45
15	0.16	0.80	2.72	0.14	0.38	1.52	0.11	0.58	1.53	0.13	0.86	2.50
16	1.56	14.61	51.83	12.28	71.00	230.58	1.45	12.89	45.08	3.47	27.59	136.97
17	0.00	0.02	0.17	0.06	0.27	1.03	0.09	0.30	16.95	0.00	0.61	5.17
18	0.00	0.00	0.00	0.00	0.00	0.00	0.03	0.00	0.00	0.00	0.00	0.17
19	0.22	2.52	16.66	2.55	21.97	105.25	0.98	10.56	85.84	0.66	6.11	46.13
20	0.09	0.42	1.64	0.14	0.16	0.31	0.06	0.52	1.64	0.13	0.56	1.48
21	0.11	0.64	1.92	0.30	0.66	1.75	0.19	0.64	2.28	0.11	0.67	1.89
22	0.22	1.47	3.81	0.44	1.20	3.70	0.23	1.27	3.97	0.28	1.33	3.64
23	0.05	0.34	0.94	0.14	0.17	0.70	1.33	12.14	73.94	0.06	0.33	1.45
24	26.86	129.34	315.89	23.67	99.48	247.72	25.25	126.94	310.22	27.91	134.39	345.58
25	0.00	0.00	0.30	0.00	0.00	0.00	0.00	0.06	0.34	0.06	0.00	0.42
26	0.03	0.16	0.28	0.02	0.05	0.27	0.05	0.23	0.98	0.03	0.28	0.63
27	0.00	0.19	0.44	0.02	0.39	0.88	0.06	0.27	3.73	0.11	0.13	2.92
28	0.05	1.86	8.08	0.00	0.00	0.09	0.33	6.38	33.81	0.13	5.50	30.44
29	0.02	0.09	1.45	0.16	6.80	30.59	0.28	6.39	30.58	0.13	2.89	12.78
30	0.00	0.00	0.17	0.05	0.00	0.17	0.64	8.77	40.30	0.00	0.28	0.86
31	0.33	1.30	5.80	4.30	32.52	84.59	8.52	58.55	227.69	8.08	54.38	213.69
32	0.34	3.52	12.83	0.63	2.97	34.56	0.73	2.11	35.41	0.59	10.23	53.84
33	0.02	0.13	0.64	0.02	0.13	0.72	0.00	0.16	0.83	0.02	0.13	0.63
34	0.02	0.09	1.30	0.08	0.44	0.75	0.03	1.02	11.41	0.06	0.06	2.19
35	0.13	1.61	11.13	0.00	0.00	0.00	0.28	7.19	34.50	0.14	5.27	33.09
36	0.00	0.06	0.33	0.00	0.06	0.27	0.11	1.34	10.00	0.00	0.05	0.50
37	0.05	0.13	0.61	0.08	0.09	0.61	0.00	0.06	0.69	0.00	0.13	0.61
38	0.00	0.00	0.34	0.05	0.00	0.14	0.02	0.00	0.31	0.00	0.00	0.23
39	0.00	0.05	0.16	0.08	0.13	0.27	0.00	0.05	0.27	0.02	0.13	0.33
40	0.02	6.64	0.09	0.00	0.16	0.16	0.00	0.16	0.00	0.03	0.06	0.09
41	0.19	1.00	1.95	0.08	0.41	1.23	0.09	10.83	42.00	0.23	1.02	2.70
42	0.16	0.83	1.31	0.16	0.77	1.50	0.19	1.11	2.16	0.27	0.94	1.61
43	0.16	0.95	3.86	0.48	2.06	6.53	0.17	0.91	2.48	0.22	1.47	3.70
44	13.08	28.11	39.45	0.13	0.14	0.47	199.86	236.06	69.61	36.25	78.47	63.83
45	0.06	1.39	7.63	0.00	0.00	0.00	0.33	8.77	32.97	0.16	5.25	27.61
46	0.00	0.13	0.53	0.08	0.22	1.30	0.02	0.38	1.08	0.00	0.08	0.52
47	0.09	3.30	26.39	0.00	2.45	11.27	0.53	9.06	44.53	0.17	4.64	41.38
48	1.92	16.78	81.48	0.05	0.08	0.06	9.17	81.80	268.95	4.28	71.53	232.80
49	0.09	0.20	0.80	0.06	0.16	0.75	1.08	5.86	4.06	0.11	0.50	2.61
50	0.03	0.22	0.63	0.05	0.45	1.14	0.02	0.09	0.66	0.02	0.02	0.17
51	0.00	0.17	0.66	0.05	0.13	0.89	0.00	0.11	0.78	0.00	0.05	0.69
52	0.02	0.25	1.09	0.17	0.09	0.53	0.08	1.11	5.66	0.02	0.17	1.48
53	0.34	3.36	11.47	0.31	1.08	4.77	0.34	1.53	4.75	0.22	1.39	5.97
54	11.86	74.11	215.81	11.44	63.31	217.22	12.66	75.08	246.59	12.98	79.83	261.73
55	1.45	12.20	49.23	11.80	70.80	229.56	1.53	12.45	45.34	4.03	32.88	113.27
56	0.48	7.63	34.66	0.00	0.08	0.00	0.53	8.38	38.88	0.56	8.14	36.59
57	1.81	16.83	41.17	0.73	49.73	202.84	1.38	11.75	32.83	2.34	36.42	171.95
58	15.08	88.03	267.13	13.38	73.38	210.70	11.02	83.45	275.88	16.30	95.63	293.28
59	0.06	0.38	1.11	0.22	0.28	0.91	0.14	0.58	0.77	0.22	1.22	2.25
60	1.69	13.22	44.84	0.97	5.06	18.22	1.83	32.80	45.75	2.00	40.33	89.44
61	1.55	12.02	42.66	11.77	64.75	212.81	1.22	10.14	55.23	3.42	27.36	148.25
62	1.20	14.25	9.73	11.83	74.30	235.39	1.08	2.23	158.00	4.09	35.42	216.16
63	0.03	0.33	1.27	0.05	0.16	0.13	0.05	0.23	1.25	0.05	0.39	1.00
64	0.50	8.42	36.61	0.75	8.83	35.50	0.53	8.97	40.92	0.52	8.73	37.61
65	0.11	0.58	2.09	0.16	0.53	1.94	0.17	1.02	3.88	0.11	0.91	6.36

## Conclusion

This paper introduces two three-term trust region conjugate gradient algorithms, TT-TR-WP and TT-TR-CG, which are capable of converging under non-Lipschitz continuous gradient functions without any additional conditions. These algorithms possess sufficient descent and trust region properties, and demonstrate global convergence. In order to assess their numerical performance, we compare them with two classical algorithms in terms of restoring noisy gray-scale and color images as well as solving large-scale unconstrained problems. To obtain highly similar SSIM and PSNR values in noisy gray-scale images, TT-TR-WP exhibits the best numerical performance in terms of running time, TT-TR-CG is the second best, MPRP is third, and A-T-PRP-A is the slowest. Furthermore, if we set the performance of TT-TR-WP as the standard, then TT-TR-CG takes around 2.34 times longer. The other algorithms take around 2.46 and 2.42 times longer, respectively. In solving the same color images, the proposed algorithms exhibit relative good performance over other algorithms. Additionally, in comparative experiments of algorithm performance, the curve of TT-TR-CG has the maximum initial value, while the curve of TT-TR-WP is the second-best, indicating that TT-TR-CG and TT-TR-WP are relatively more robustness and have high stability when facing diverse situations. In summary, TT-TR-WP and TT-TR-CG exhibit relatively better performance in terms of applicability and robustness.

## Data Availability

All images are sourced from published papers or the internet, and there are no copyright disputes. All data generated or analysed during this study are included in this published article [and its supplementary information files].
